# Standalone GPS L1 C/A Receiver for Lunar Missions

**DOI:** 10.3390/s16030347

**Published:** 2016-03-09

**Authors:** Vincenzo Capuano, Paul Blunt, Cyril Botteron, Jia Tian, Jérôme Leclère, Yanguang Wang, Francesco Basile, Pierre-André Farine

**Affiliations:** École Polytechnique Fédérale de Lausanne (EPFL), Electronics and Signal Processing Laboratory (ESPLAB), Rue de la Maladière 71b, CH-2002 Neuchâtel 2, Switzerland; paul.blunt@epfl.ch (P.B.); cyril.botteron@epfl.ch (C.B.); jia_epfl_tian@163.com (J.T.); jerome.leclere@lassena.etsmtl.ca (J.L.); wggaily@163.com (Y.W.); basile.fran1989@gmail.com (F.B.); pierre-andre.farine@epfl.ch (P.-A.F.)

**Keywords:** GNSS, GPS, orbital filter, space navigation, Kalman filter, lunar mission, signal processing

## Abstract

Global Navigation Satellite Systems (GNSSs) were originally introduced to provide positioning and timing services for terrestrial Earth users. However, space users increasingly rely on GNSS for spacecraft navigation and other science applications at several different altitudes from the Earth surface, in Low Earth Orbit (LEO), Medium Earth Orbit (MEO), Geostationary Earth Orbit (GEO), and feasibility studies have proved that GNSS signals can even be tracked at Moon altitude. Despite this, space remains a challenging operational environment, particularly on the way from the Earth to the Moon, characterized by weaker signals with wider gain variability, larger dynamic ranges resulting in higher Doppler and Doppler rates and critically low satellite signal availability. Following our previous studies, this paper describes the proof of concept “*WeakHEO*” receiver; a GPS L1 C/A receiver we developed in our laboratory specifically for lunar missions. The paper also assesses the performance of the receiver in two representative portions of an Earth Moon Transfer Orbit (MTO). The receiver was connected to our GNSS Spirent simulator in order to collect real-time hardware-in-the-loop observations, and then processed by the navigation module. This demonstrates the feasibility, using current technology, of effectively exploiting GNSS signals for navigation in a MTO.

## 1. Introduction

GNSS receivers have been successfully adopted as key elements of the navigation systems for many space borne missions. Typically, they are used in LEO where the availability of the GNSS signals is very good and high accuracy positioning can be achieved. Using a GNSS receiver as the core navigation system enables greater autonomy of a spacecraft and reduces the burden and costs of network operations [1]. For this reason, the autonomous navigation capabilities of GNSS are extremely appealing, not only for LEO but for higher altitude Earth orbits as well. It is even possible for spacecraft orbiting at altitudes beyond the GNSS constellations to receive weak signals from the opposite side of the Earth. These receivers are picking up the spillover of the main lobe of the GNSS transmit antenna pattern around the Earth mask and also signals from the weaker side lobes of the antenna pattern. Research presented in [2,3,4,5,6,7,8] shows feasibility studies of GNSS as navigation system for space missions in High Earth Orbits (HEO) and beyond up to Moon altitude. Other research work, such as [9,10,11,12] and [13] has proposed GNSS receivers designed for such missions and the demonstration of a receiver positioning in HEO is detailed in [14]. In [15,16,17] experimental demonstrations are described that show the GNSS signals availability even at higher orbits than GEO. Research continues to investigate the potential of using GNSS for lunar missions, in particular for Earth-Moon transfer orbits.

In this paper, we describe the GPS L1 C/A hardware receiver, named “*WeakHEO*” receiver, which we have designed and developed as a proof of concept for lunar missions at the ESPLAB EPFL. Firstly, the hardware architecture of the receiver is presented followed by a description of the defined mission and expected GNSS signal environment in which the receiver has to operate. The high-sensitivity acquisition and tracking algorithms of the receiver are then described before concluding with navigation test results from the receiver.

## 2. The *WeakHEO* Receiver Architecture

**Figure 1:**
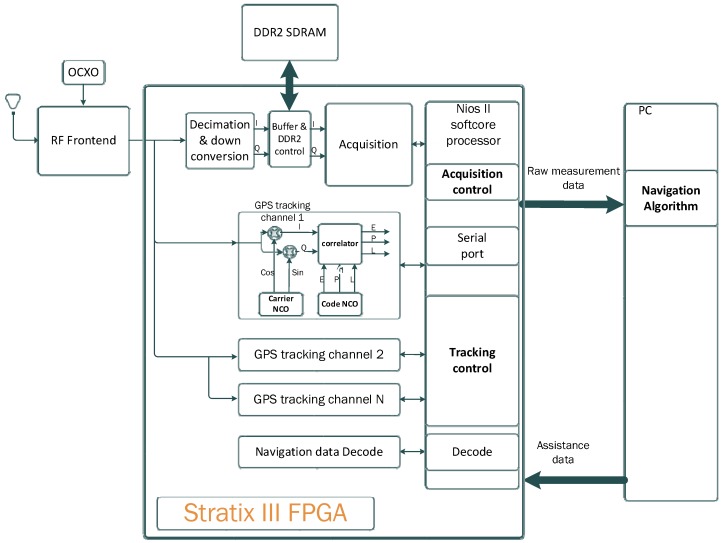
Architectural components of the *WeakHEO* receiver.

The architecture of the *WeakHEO* receiver is shown in Figure 1. The system comprises of three main elements as follows.

- **A tri-band (L1, L2, L5) RF front end**, which amplifies, filters and down converts the GNSS signals to an intermediate frequency (IF) where they are sampled. The reported initial implementation is focused on processing and utilisation of the GPS L1 C/A code signal. However, a triple frequency L1, L2, L5 front end (an early version of the front end in [18]) was selected to allow future expansion to these frequencies. A high sampling rate is used to enable the receiver to support precision tracking architectures and additional wider bandwidth signals in the future. A common IF frequency of 53.78 MHz is used for all three bands and signals are sampled at 40.96 MHz with 4-bit resolution. The RF front is driven by a stable, low-phase noise Oven Controlled Crystal Oscillator (OXCO).
- **A DE3 FPGA platform**. An FPGA platform was required to allow custom designs of the acquisition and tracking engines within the receiver. The *WeakHEO* receiver uses the same development platform and builds on the FPGA-based architecture of the “Signature” receiver developed by ESPLAB in EPFL [19]. Its core component is a Stratix III FPGA (Field Programmable Gate Array) receiving the parallel sampled data from the RF front end. The FPGA contains a softcore NIOS II (32bit RISC) processor and performs all the high sensitivity acquisition, tracking and navigational data decoding processes. Raw measurements (pseudoranges, pseudorange rates, signal parameters, time, *etc.*) are passed to the PC through a UART interface at a rate of 0.1 Hz. Note that this rate has been chosen in order to allow the real-time processing of the navigation solution on the PC (currently programmed in Matlab). In the next version of the receiver, a faster update rate will be selected. An external memory (DDR2 SDRAM) connected to the FPGA is also used as buffer for the acquisition.
- **A PC**, The PC then performs the navigation solution in real time or can record and compute the orbital filter calculations offline.

### 2.1. Operations of the Receiver

The operations of the receiver are performed in several steps as follows:

(1) The navigation software on the PC determines which GPS satellites are visible and estimates the Doppler for each satellite. This information is then sent to the FPGA through the UART interface. This allows a reduction of the frequency search space for the acquisition thus a reduction of the acquisition time.
(2) The acquisition searches the satellites in view within a frequency search space around the coarse Doppler given. Once a satellite is acquired, there is a transition phase before the tracking to determine the position of the bit edge. Following this, tracking is started and the Time Of Week (TOW) is decoded from the received navigation data.
(3) The measurements (pseudoranges, pseudorange rates, satellite PRN, estimated C/No and TOW) are sent to the computer by the FPGA at a rate of 0.1 Hz and a PVT solution is computed.

### 2.2. Description of the Mission Scenario

**Figure 2:**
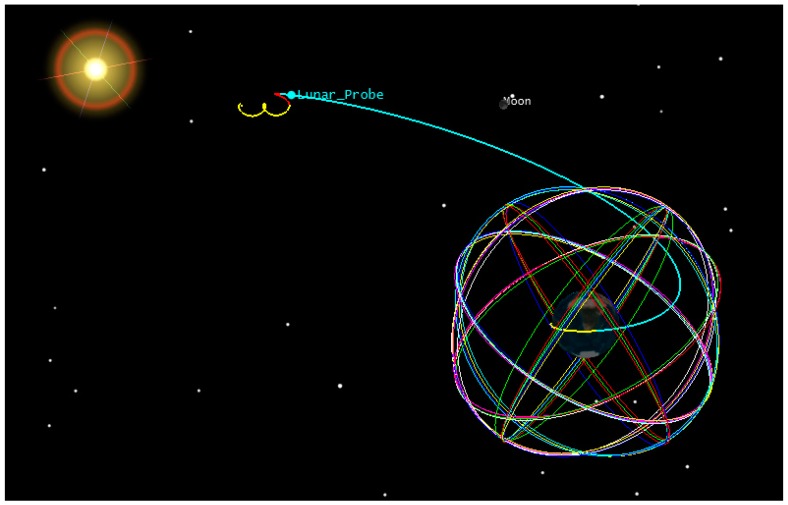
STK representation of the considered MTO.

**Figure 3:**
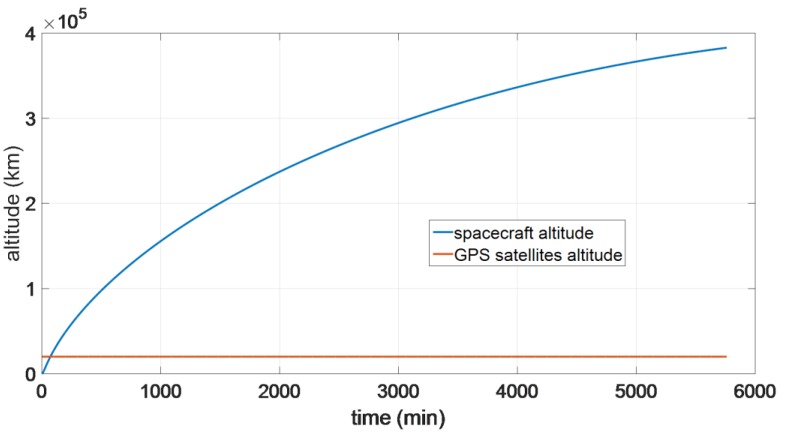
Relation between altitude and time during the considered MTO and GPS constellation altitude.

The reference trajectory for our study is a direct Earth-Moon Transfer Orbit (MTO). The initial position and velocity with the respect to the Earth Centered Inertial frame (ECI) are reported in Table 1, as well as some characteristics assumed for the host spacecraft. The full trajectory is propagated from the initial conditions by the PosApp software of our Spirent GSS8000 simulator, taking into account gravitational effects from the Earth, Sun and Moon, atmospheric drag and the solar radiation pressure. The reference trajectory is represented by the blue portion of the MTO shown in Figure 2. The reference orbit has been validated using the System Tool Kit (STK) [20]. Figure 3 shows the relation between altitude and time during the considered MTO and also the GPS satellites altitude.

### 2.3. GNSS Signal Characteristics

#### 2.3.1. Simulation Models and Assumptions

In our simulations we have modelled a GPS constellation consisting of 32 GPS satellites, allocated in the six orbital planes according to [21]. Realistic GPS L1 C/A signals are generated by our Spirent GSS8000 full constellation simulator, where the signal power level at the receiver $P_{r}$ is modelled as [22]:

(1) $$P_{r}=P_{ICD}+O_{G}+20\times \log_{10}\left(\frac{R_{0}}{R}\right)-L_{tx}-L_{rx}$$

where:

$P_{ICD}=-128.5\;dBm$is the guaranteed minimum signal level for the GPS L1 C/A signals on the Earth according to [21].$O_{G}=+3\;dB$is the global offset, chosen to obtain in simulation the performance obtained when real signals are received. Typically, the transmitted signal power levels are from 1 to 5 dB higher than the minimum received ones [23], for this reason an intermediate value of 3 dB has been chosen.$R_{0}$is the reference range used for inverse-square variation calculation and equal to the range from a receiver to the GNSS satellite at zero elevation.Ris the range from GNSS satellite to the receiver.$L_{tx}$is the loss from the GNSS satellite transmit antenna in the direction of the receiver. 3D GPS antenna patterns have been modelled, as described in our previous study [24,25].$L_{rx}$is the loss from the receiver antenna in the direction of the GNSS satellite.

#### 2.3.2. Signal Power and Dynamics

**Figure 4:**
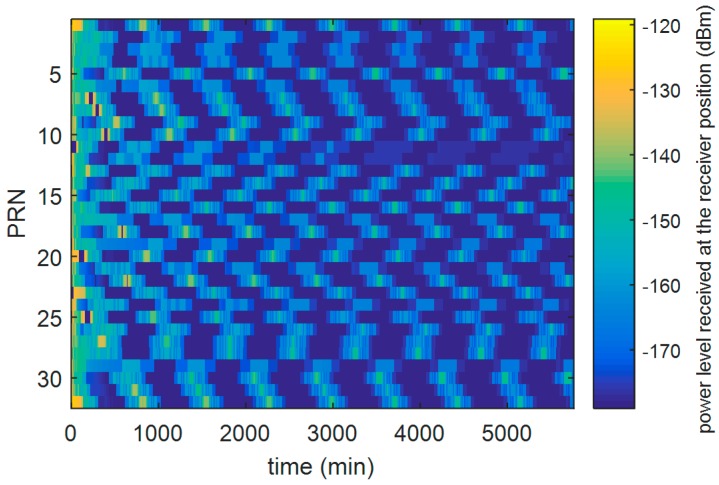
Received power levels of the GPS L1 C/A signals as a function of the time, during the full considered trajectory, by assuming a 0 dBi receiver antenna gain.

**Figure 5:**
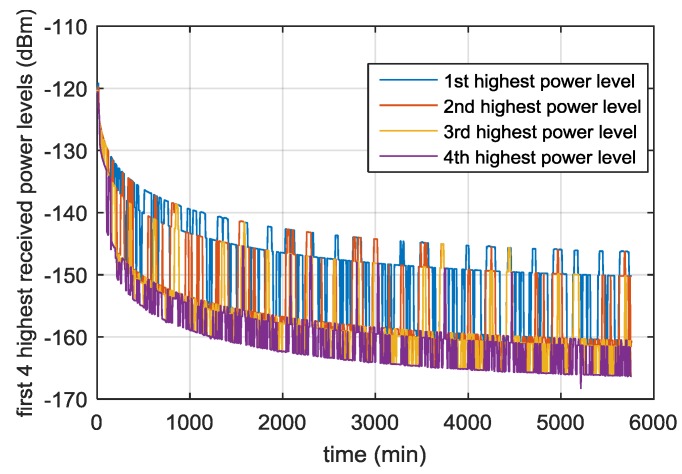
First, second, third and fourth highest received power levels of the GPS L1 C/A signals as a function of the time, during the full considered trajectory, by assuming a 0 dBi receiver antenna gain.

**Figure 6:**
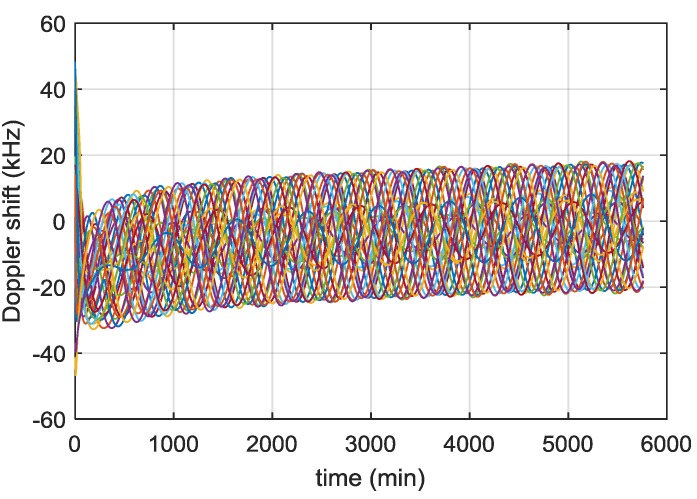
Doppler shift of received GPS L1 C/A signals across the MTO.

**Figure 7:**
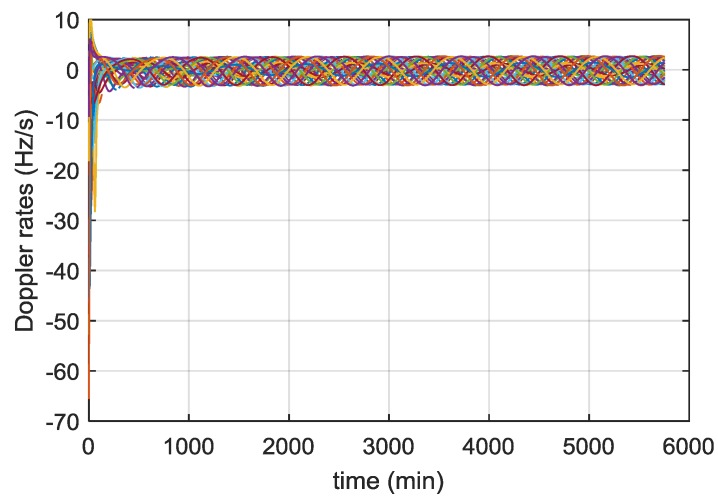
Doppler rates of received GPS L1 C/A signals across the MTO.

In this section we report some significant simulation results from our previous studies [13,24], and [26] used to define the main receiver characteristics. Figure 4 shows the received power levels of all the GPS L1 C/A signals as a function of the time, during the full considered trajectory. As shown in Figure 5, the fourth strongest GPS L1 C/A signal (at least four ranges are required to compute the 3D position) at receiver antenna reaches approximately a minimum power level of −168.5 dBm. For this reason, a very high sensitivity receiver architecture is required. Moreover, the carrier frequency is affected by Doppler shifts and Doppler rates (see Figure 6 and Figure 7, respectively) of up to 20 kHz and up to 4 Hz/s respectively at the Moon altitude (where the signals are weaker) and of up to 60 kHz and up to 65 Hz/s at the beginning of the MTO (where the signals are stronger), requiring a certain robustness against high dynamics for the receiver.

The identified minimum power level of −168.5 dBm of the fourth strongest signal during the full considered MTO can be seen as a power sensitivity value required for the receiver to acquire and track at least four signals (and then provide the navigation solution) with a certain probability. Then, assuming a receiver antenna gain of 10 𝑑𝐵𝑖, we have targeted a sensitivity of at least −159 dBm, slightly higher than the minimum required.

According to [27], it is possible to calculate the carrier-to-noise density ratio C/N_0_ (in dB-Hz) from the received power $P_{r}$ (in dBm) by using the following formula, valid for a front-end noise figure of 2 dB and an effective antenna temperature of 130 K:

(2) $$C/N_{0}=P_{r}+174$$

A received signal level of −159 dBm then corresponds to a C/N_0_ of 15 dB-Hz.

However, when a GNSS simulator is used, the effective antenna temperature is the room temperature, which increases the noise. In that case, the carrier-to-noise ratio can be obtained as

(3) $$C/N_{0}=P_{r}+172$$

A received signal level of −157 dBm then corresponds to a C/N_0_ of 15 dB-Hz for testing with the GNSS simulator.

### 2.4. Geometric Dilution of Precision (GDOP) and Ranging Errors

As described in [24] in detail, the achievable navigation accuracy at Moon altitude is strongly limited by a very poor relative geometry between the receiver and the transmitters (very large GDOP values) and large ranging errors. For instance, at Moon altitude, simulations in [26] show GDOP values much higher than 1000 for an acquisition sensitivity of 15 dB-Hz. Due to the very low carrier to noise ratio $C/N_{0}$ values, when processing GPS L1 C/A signals, the code tracking thermal range jitter $\sigma_{tDLL}$ can achieve values larger than 6 m [24]. A detailed characterization of GDOP and ranging error for different sensitivity values and different GNSS signals is provided in [24].

### 2.5. GPS Acquisition

#### 2.5.1. Acquisition Strategy

The three main acquisition methods for GNSS signals are the serial search (SS), the parallel frequency search (PFS), and the parallel code-phase search (PCS) [28]. Compared to the PCS and the PFS methods, the acquisition time of the SS method is extremely long, therefore, the SS was not considered. Compared to PCS, PFS has two main drawbacks. Firstly, an extra loss due to the integration before the fast Fourier transform (FFT) and secondly a loss due to the mismatch between the replica code chipping rate and the received code chipping rate, especially important for long integration times [28]. Since the sensitivity is of prime importance in high altitude space applications, these additional losses are not acceptable, and therefore the PCS technique was selected.

**Figure 8:**
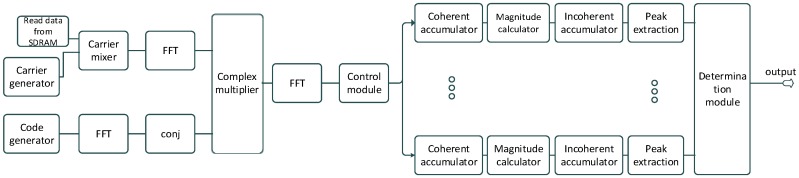
Block scheme of the acquisition module.

The acquisition structure of receiver is illustrated in Figure 8. In acquisition, the receiver uses coherent accumulations, which are the length of a full navigation data-bit (20 ms for GPS C/A code). The results are then non-coherently accumulated to gain further sensitivity. To reduce the effect of the data-bit transitions a number of different accumulations are formed with different starting points. Ideally, for GPS C/A code we would have 20 accumulations spaced 1 ms apart. However, to limit the impact on the FPGA resources we form 10 accumulations spaced 2 ms to achieve a balance between performance and the available resources.

Following the results of Section 2.3.2, the sensitivity targeted is 15 dB-Hz, slightly higher than the minimum required to acquire at least four signals. Using the methodology proposed in [27], the theoretical analysis of the acquisition parameters is presented in Table 2. This is used to estimate the total required integration time required for a successful acquisition. In acquisition, the receiver’s sampling rate of 40.96 MHz is decimated to 4.096 MHz as a compromise to improve processing performance. The resolution in the code search is one sample so this results a resolution around ¼ of a chip. A higher sampling frequency requires more samples to be processed but results in a fine resolution in the code search domain. A lower frequency means less samples to process but poorer resolution with higher losses from the code misalignment. In tracking the full sample rate of 40.96 MHz is used to allow precision tracking from narrow correlator spacings. A frequency step of 25 Hz has been selected, again as compromise for the performance. Finally, the maximum tolerable Doppler rate error is defined as the Doppler rate, which implies a shift of one frequency bin during the integration time.

#### 2.5.2. Acquisition Hardware Implementation

**Figure 9:**
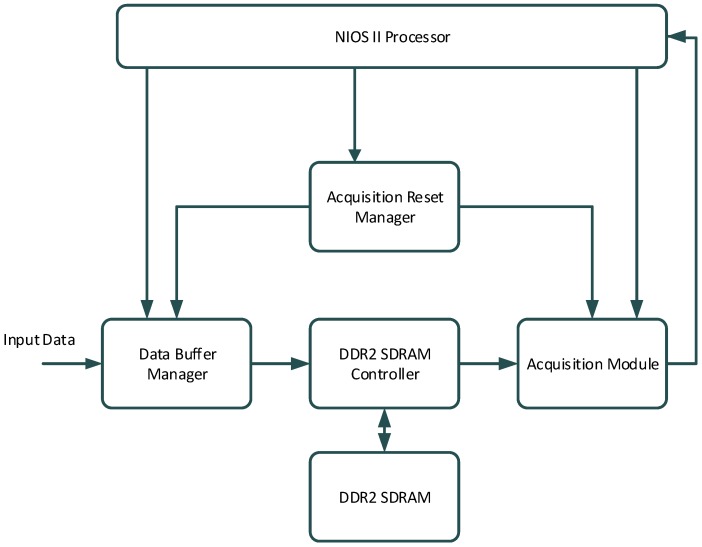
Global structure of the acquisition implementation.

The FPGA implementation of the acquisition is shown in Figure 9. The Nios II processor is used to manage and configure the different blocks (e.g., the acquisition module is configured with parameters such as Doppler frequency, PRN code, number of integrations, *etc.*), and to analyze the data provided by the acquisition module to decide if a signal has been detected. The acquisition module processes the input data using the PCS method as shown in Figure 8 and provides acquisition statistics (peak value, mean value, standard deviation, *etc.*) to the Nios II processor. Since the integration time is very long (9.5 s), the amount of data to save is significant (around 39 M samples). Therefore, a DDR2 SDRAM external to the FPGA is used to save this data.

In high sensitivity acquisition, the acquisition time can be very long and the transition between the acquisition and the tracking becomes difficult. Thus, we propose to acquire the signal twice, reducing the frequency search space after the first step.

The time spent to search one frequency bin is defined as:

(4) $$T_{FB}=\frac{f_{S}T_{I}}{f_{FPGA}}=\frac{4.096\times 10^{6}\times 9.5}{163.84\times 10^{6}}=237.5\;\mathrm{ms}$$

where *f_S_* is the sampling rate, *T_I_* is the total integration time, and *f_FPGA_* is the clock frequency of the FPGA. For the first acquisition, the frequency search space considered is ±35 kHz. With a frequency step of 25 Hz, there are *N_FB_*_,1_ = 2801 frequency bins, thus the time to search the entire frequency search space is:

(5) $$T_{A,1}=T_{I}+N_{FB,1}T_{FB}=9.5+2801\times 237.5\;10^{-3}=674.5\;s$$

According to Figure 7, the Doppler rate is less than 4 Hz/s after the initial few hours of the MTO. Therefore, during the first acquisition the frequency of the received signal may have shifted by up to 2700 Hz (4 Hz/s × 675 s). For the second acquisition, the frequency search space is then ±2700 Hz, which corresponds to *N_FB_*_,2_ = 217 frequency bins. The time to search the frequency search space of the second step is:

(6) $$T_{A,2}=T_{I}+N_{FB,2}T_{FB}=9.5+217\times 237.5\;10^{-3}\approx 61\;s$$

During this time, the frequency of the received signal could have shifted by up to 244 Hz, which is still too large to start correctly the tracking. However, provided we recorded the time between the two consecutive acquisitions, the Doppler rate can been estimated by looking at the evolution of the Doppler frequency, or from the evolution of the code delay. From Figure 7, we can assume the Doppler rate is constant during a short time interval and therefore it is possible to initialise the tracking from these results.

#### 2.5.3. Acquisition Aiding from Navigation

For a standalone receiver, some aiding can still be obtained from the navigation result. The *a priori* knowledge consists of:

- Position (from the last known position stored in memory),
- Time (from the real-time clock),
- Reference frequency (since the receiver oscillator offset is determined by the navigation solution)
- Approximate GNSS satellite positions and velocities (calculated from the almanac data stored in memory).

Therefore, the frequency search uncertainty for the acquisition mainly depends on the time uncertainty, receiver velocity and position uncertainties, and almanac uncertainty, which will be discussed below.

##### Time Uncertainty

By time, we mean the time of week (TOW) from the navigation data. This can be delivered as the GPS week and seconds of the week. Provided the receiver is tracking at least one satellite above 15 dB-Hz the time can be decoded and considered to be known within 1 s. The rate of change of the Doppler frequency is up to 4 Hz/s according to Figure 7. Therefore, for 1 s of error in time, the corresponding error in the Doppler estimation is up to 4 Hz. Once a navigation fix is achieved, as we are using a stable OCXO our time knowledge will not drift quickly in periods of poor GNSS visibility (around 1 ms/day) and so is likely to be much better.

##### Receiver Velocity Uncertainty

According to our post processing simulations, whose assumptions are described in [25], when the GPS solution is not filtered through any orbital forces model, the receiver velocity uncertainty can reach approximately 700 m/s at Moon altitude, that means that the corresponding frequency uncertainty is 700 m/s/0.19 m = 3684 Hz (0.19 m being the wavelength of the L1 C/A signal).

##### Receiver Position Uncertainty

According to our simulations, when the GPS solution is not filtered through any orbital forces model, the receiver position uncertainty can reach peaks of 80 km at Moon altitude (neglecting a very few higher peaks). Using the equations given in Section 3.6.5 of [27], the corresponding frequency uncertainty is (3800 m/s × 80 km)/(380 000 km × 0.19 m) = 4.2 Hz, where 3800 m/s is the GPS satellite speed, 380,000 km is the distance between the Earth and the Moon, and 0.19 m is the wavelength of the L1 C/A signal.

##### Almanac Uncertainty

The precision of the ephemeris is very high, so the frequency uncertainty induced by the ephemeris can be ignored. According to Section 3.6.6 of [27], if the almanac data are used for frequency assistance, the frequency uncertainty is 60 Hz.

##### Total Search Uncertainty

According to the analysis above, the total frequency uncertainty is about ±3692 Hz. Note that for the position and velocity uncertainty, to be conservative we have considered the worst case that is obtained at Moon altitude; in fact it is the highest altitude of the trajectory and it corresponds to the highest GDOP and accordingly to the highest position and velocity errors.

### 2.6. GPS Tracking

#### 2.6.1. Bit Synchronisation and Navigation Data Decoding from very Weak Signals

Following the acquisition stage, the acquisition engine passes its estimates of Doppler, Doppler rate, code offset and bit position to the tracking channel and is then free to look for the next satellite. The position of the navigation data bit edge must be located and confirmed. This is necessary as although the acquisition uses full navigation data bit length accumulations of 20 ms, it only computes 10 branches spaced 2 ms apart to reduce complexity. In the tracking channel 20 branches, spaced 1 ms apart are then formed and accumulated to confirm the bit edge position. During this process the tracking channel operates a low bandwidth FLL (0.2 Hz) initialised with the estimation of Doppler, Doppler rate, code offset and bit position from the acquisition engine. Following confirmation of the bit edge position, decoding of the navigation data bits is started. The theoretical probability of a navigation data bit error of the C/A code signal assuming carrier phase tracking is given by [23]:

(7) $$P_{B}=\frac{1}{2}erfc\left(\sqrt{c/n_{0}\tau_{a}}\right)$$

where erfc is the complementary error function, $c/n_{0\;}$is the carrier to noise density ratio (scalar) and $\tau_{a}$ is the accumulation time (20 ms for C/A code). The probability of a bit error is 0.13 at a $C/N_{0}$ of 15 dB-Hz and 0.056 at 18 dB-Hz. A $C/N_{0}$ of 27.5 dB-Hz is required to give a probability of bit error of 10^−6^. As each subframe is 300 bits long, this probability would result in less than one contaminated subframe in every 3000. However, as shown in [29,30], the fact that the message is regularly repeating can be exploited to effectively increase the accumulation time and reconstruct the message at weaker signal levels.

**Figure 10:**
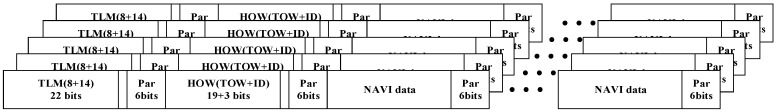
GPS frame structure.

The data decoding procedure can be divided into two steps, the first step is frame synchronization (finding the preamble), the second step is to decode the desired data from multiple subframe repetitions. As depicted in Figure 10, each GPS frame contains 5 sub-frames. Every sub-frame has 6 s duration. It starts with a known 8 bit preamble and consists of 10 words. Each word has 30 bits. The preamble marks the beginning of each sub-frame and is repeated every 6 s.

**Figure 11:**
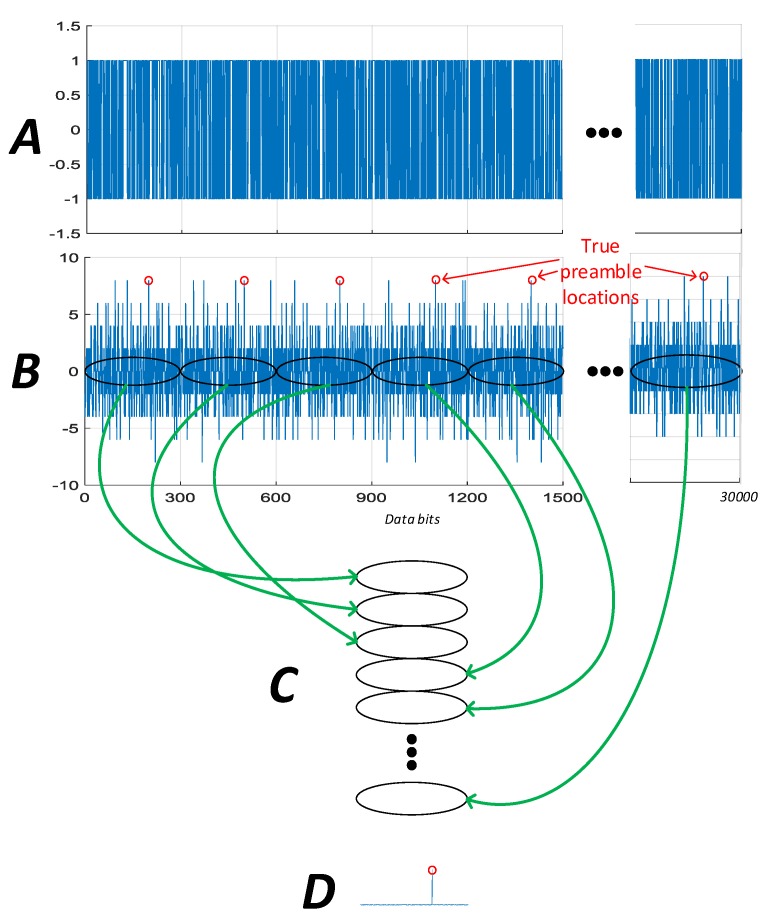
GPS preamble location.

The following steps are used to find out the position of the preamble from received data (see also Figure 11).

(1) Store 20 frames (*i.e.*, 20 × 30 s, or 100 sub-frames, *i.e.*, 30,000 bits) of original demodulated data in a vector ***A***.
(2) Search for all the preamble-like sequences. Find all the likely preambles of vector ***A***, and store these correlation values in vector ***B***. Due to the possible 180° phase ambiguity induced by phase tracking and the chance of cycle slips with weak signals, positive and negative correlations are searched for.
(3) Matrix ***C*** is generated by reshaping the correlation vector ***B*** into sub-frame length rows. Each column then represents a possible preamble location in the sub-frame. The size of this matrix is 300 × 100. The absolutes values of matrix ***C*** are then accumulated down the columns to form a vector ***D***.
(4) The three largest correlations of vector ***D*** are recorded. The largest value should be the correct position of the preamble, however, with weak signals other possible locations may need to be checked.
(5) The first position is assumed correct, the vector ***A*** reshaped into a matrix of sub-frame length rows and the sub-frame number from each successive sub-frame is checked after column wise accumulation. If the sub-frame number is incrementing correctly, this position is declared correct and used in subsequent processing. Otherwise, the other probable positions of the preamble are checked.

Assuming the signal is tracked consistently throughout, after preamble correlation and column wise accumulation, the right position of preamble will be found and the sub-frame will be synchronized.

Once the subframe is synchronised it is possible to reconstruct the desired data items from the stored data by accumulation down the column. The parity of each word in the matrix can then be checked individually. If the parity check is passed, the word is decoded and its position marked. The choice of 20 entire navigation frames is made so that sub-frames 1 to 3 will be repeated 20 times resulting in a probability of bit error in the accumulated data of less than 10^−6^ at 15 dB-Hz. It is likely that time and ephemeris parameters can be provided by the platform and only frame synchronisation is required. Therefore, we have only decoded the time of the week in our hardware implementation to demonstrate the principle.

**Figure 12:**
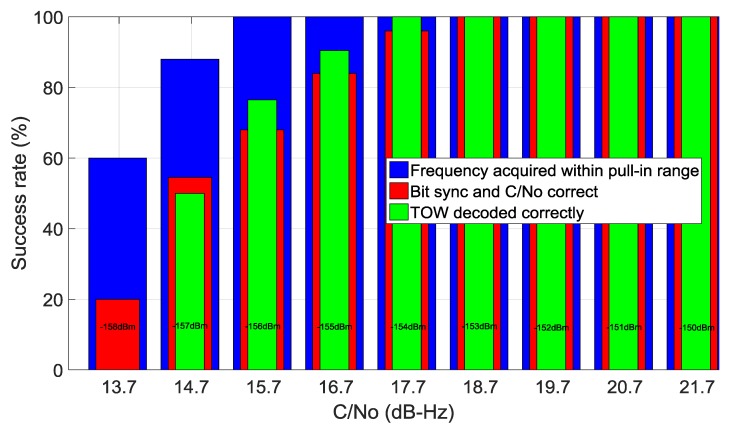
Sucess rate of acquisition stages with varying signal levels.

The success rate of the acquisition engine with signals of different carrier to noise density ratios (C/No) is shown in Figure 12. Here 25 trials were performed at each signal level and the success rate recorded for the acquisition, bit synchronization, and data decoding stages. The signals were generated with our Spirent GSS800 simulator and the output power is displayed in Figure 12. The decoding of the time of the week is used to determine if frame synchronisation was successful. Using typical derivations from [23,31] the detection threshold is chosen such that the probability of detection is theoretically 0.95 at 15 dB-Hz and probability of false alarm is 10^−3^.The acquisition results of Figure 12 are slightly worse but within 1 dB of the expected values. Clearly, the receiver is limited by its ability to synchronise and decode the navigation data before the acquisition limit is encountered. Despite this, at 15 dB-Hz the receiver is still able to acquire and decode the navigational data with a success rate of around 60% for each attempt.

#### 2.6.2. Weak Signal Tracking

The *WeakHEO* receiver is assumed to be a stand-alone receiver with a low rate communication interface to the spacecraft platform. Therefore, knowledge of the full navigational data sequence is not assumed and data wipe-off is not used. Data wipe-off allows the use of pure (non-squaring) PLL discriminators to be used in tracking for lower jitter and a larger pull-in range compared to Costas-type discriminators.

**Figure 13:**
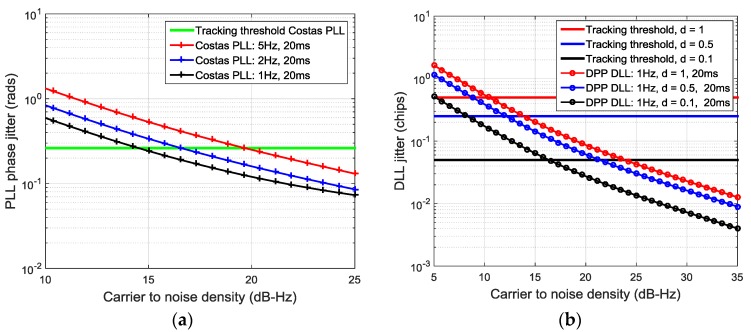
*(***a**) PLL jitter; (**b**) DLL jitter *versus* C/No.

Typical expressions for the jitter of conventional GNSS tracking loop can be found in [23,32]. Figure 13a shows the expected PLL jitter and a conservative loss of lock threshold for a third order Costas PLL, assuming thermal and oscillator noise (based on the *WeakHEO* OCXO) are the only error sources and an integration time of 20 ms. This indicates that in the presence of navigation data flips we need to use bandwidth as low as 1 Hz to operate at 15 dB-Hz. The code tracking loop of the receiver is a first order loop aided by the carrier loop and uses a bandwidth of 0.1 Hz. Figure 13b shows the DLL jitter for the dot-product power discriminator with a 0.1 Hz, a 20 ms integration time and different early-to-late correlator spacing *d*. Correlator spacings of 0.25 chip or greater are required to maintain code tracking at 15 dB-Hz. If an adaptive spacing is used, smaller correlator spacings can be used at higher signal levels to reduce the pseudorange jitter. It should be noted that using such low bandwidths to improve the performance in weak signal environments lengthens the time between statistically independent measurements from the code and carrier loops [32]. This is not critical for this application. These low bandwidths are suitable for operation at altitudes significantly above the GNSS constellation; however, during the first part of the MTO the line-of-sight jerk can be as high as 6 ms^−3^. The received signal strength is far greater at this point but still requires a carrier loop PLL bandwidth of around 5 Hz to tolerate this jerk [32]. Therefore, when conventional approaches to GNSS tracking are used for this application the carrier loop bandwidth will need to be adjusted during the MTO.

Conventional tracking models the incoming system as having deterministic dynamics by typically using fixed tracking loop bandwidths. An alternative method, which provides more flexibility is based on Kalman filter theory. Rather than assuming deterministic dynamics, the Extended Kalman Filter (EKF) assumes that the signal dynamics follow a linear stochastic model. This allows an adaptive scheme where the response to dynamics is adjusted based on the signal conditions. Therefore, the ranging processor is designed to adapt the tracking loop bandwidths as a function of the measured *C/N_0_*, to maintain the optimum trade-off between noise resistance and dynamics response. It should be noted that this is not exclusively possible with the EKF based tracking and can be applied to conventional tracking. However, this is performed implicitly when using the EKF approach as part of its dynamic model.

A number of variations on EKF tracking have been developed for both code and carrier tracking [29,33,34,35]. Comparing EKF tracking to conventional architectures is troublesome as the non-linear models used in the EKF implementation are effectively constantly adapting its tracking bandwidth. Generally, jitter comparisons are made with conventional architecture under different dynamic conditions. However, this does provide to opportunity to choose scenarios and tracking loop settings to exaggerate the benefits of one technique over another. An attempt to compare the techniques by experimentally determining the steady-state bandwidth of the EKF and finding the equivalent performing PLL is found in [36]. Here, the EKF is demonstrated to be able to track the carrier phase down to 10 dB-Hz, around a 7 dB improvement under equivalent conditions. Tests with software receiver implementations of conventional and EKF tracking in [37] show around a 4 dB to 7 dB improvement in sensitivity for the EKF approach.

The EKF is well suited to applications such as the one considered here where the platform has predictable dynamics and the receiver’s oscillator can be measured and modelled. For the *WeakHEO* receiver we have implemented a first order EKF for carrier phase tracking as detailed in [29]. The EKF is only used for the carrier tracking and conventional tracking is used for the code loop to minimise the computational load.

In [26] we show how Doppler and Doppler rate aiding from an integrated orbital filter can be used to improve sensitivity of conventional FLL tracking to around 11 dB-Hz. This is considered suitable for a stand-alone receiver as the orbital filter can be integrated into the receiver’s navigation software.

**Figure 14:**
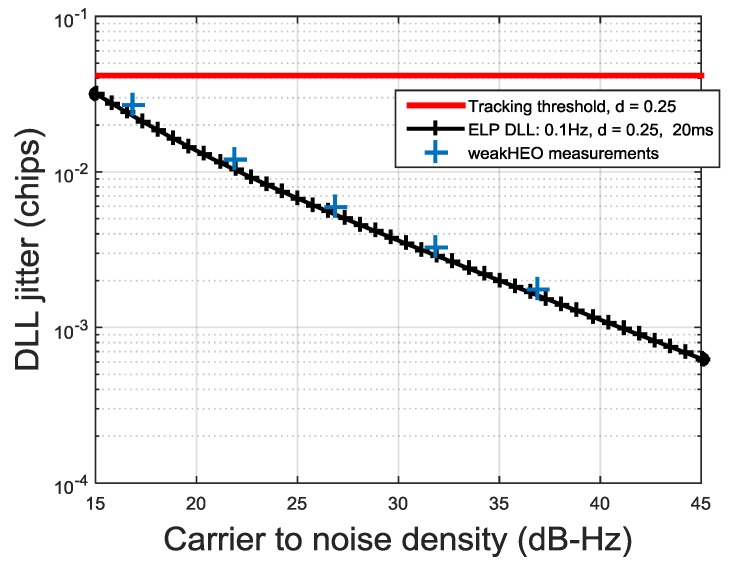
*WeakHEO* DLL jitter *versus* C/No.

Figure 14 shows double difference measurements of the *WeakHEO* DLL jitter taking with our GNSS simulator with varying carrier to noise density ratios. Reasonable agreement with the theoretical values can be found.

### 2.7. GPS Navigation

An orbital filter has been implemented that integrates the GPS observations with an orbital forces model, by means of an adaptive Extended Kalman filter (EKF). The EKF integrates the least square single-epoch position and velocity solutions that are computed using the pseudoranges and the pseudorange rates output by the *WeakHEO* GPS receiver, together with the position and velocity predicted by an orbital forces model. The resultant filtered navigation solution can efficiently mitigate the effect of the very high GDOP and large ranging errors; indeed, it is significantly more accurate than the unfiltered least square single-epoch solution, as shown in Section 3. Thus, it can be used to aid the GPS receiver to acquire and track weak GPS signals while experiencing high dynamics. A detailed description of the orbital filter and its aiding to the acquisition and tracking modules can be found in [26]. In a different configuration, less simple but potentially more efficient, the EKF can integrate directly pseudoranges and pseudorange rates provided by the tracking loops together with their prediction provided by the orbital forces model. This has been investigated in our previous study [25], which also describes the performance of our orbital filter when it uses signals transmitted by the GPS-Galileo combined constellation. A comparison of the two configurations is described in [38].

As described in [24], during a MTO, once the receiver is above the GPS constellation, the GPS accuracy strongly decreases with the altitude. Both pseudorange error and GDOP worsen as the receiver goes further from the GPS constellation. For this reason, if the covariance matrix of the measurements *R* is composed of constant variance values over time, the filter cannot be tuned properly during the full MTO (Figures 6 and 7 of [38] shows the effect of modelling *R* as constant matrix).

**Figure 15:**
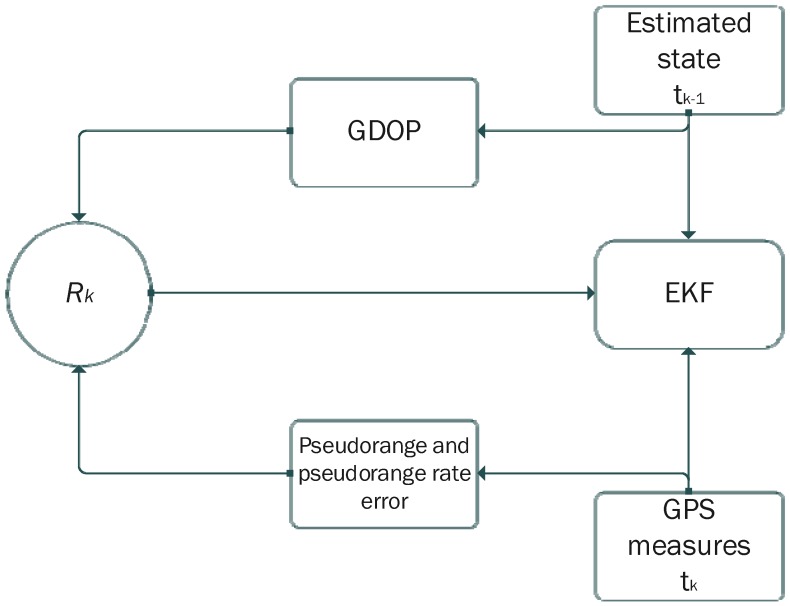
Dynamic tuning of the covariance matrix of the measurements.

As described in [38], the covariance matrix *R* of the filter has to be adapted to the GPS measurements accuracy. Indeed, the *R* matrix is modelled as function of the GDOP multiplied by the estimated pseudorange error. The adaptive strategy is illustrated in Figure 15. From the estimated state computed at the previous time-step, the filter estimates the GDOP and the noise on both pseudorange and pseudorange rate measures to update the covariance matrix *R*.

## 3. *WeakHEO* Navigation Performance

In order to test the achievable performance in terms of position and velocity determination we have selected two representative portions of the MTO, each of one-hour duration.

**Figure 16:**
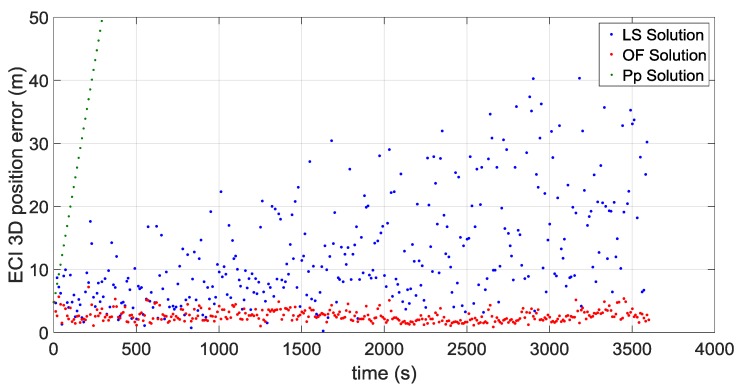
3D position error for the considered portion which starts at GEO altitude for: the single-epoch least square (LS) solution (in **blue**); the orbital filtered (OF) solution (in **red**); and the propagated (Pp) solution (in **green**).

**Figure 17:**
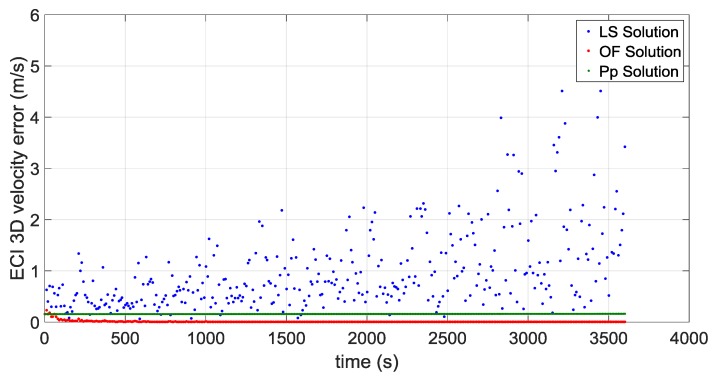
3D velocity error for the considered portion which starts at GEO altitude for: the single-epoch least square (LS) solution (in **blue**); the orbital filtered (OF) solution (in **red**); and the propagated (Pp) solution (in **green**).

**Figure 18:**
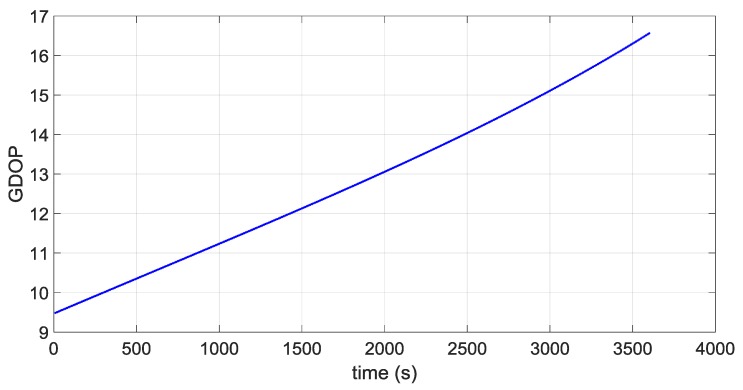
GDOP during the MTO portion that starts at 36,000 km altitude.

**Figure 19:**
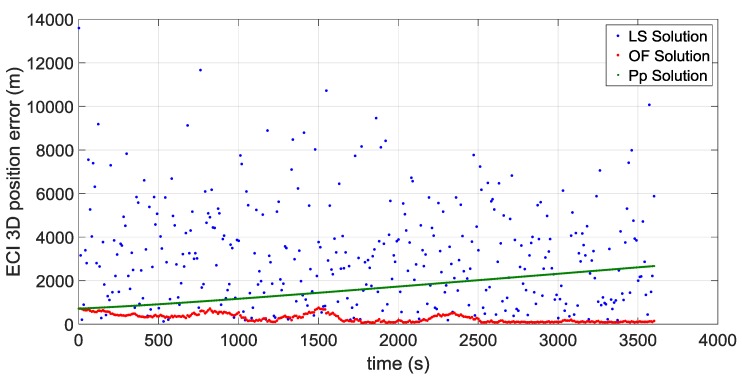
3D position error for the considered portion at Moon altitude for: the single-epoch least square (LS) solution (in **blue**); the orbital filtered (OF) solution (in **red**); and the propagated (Pp) solution (in **green**).

**Figure 20:**
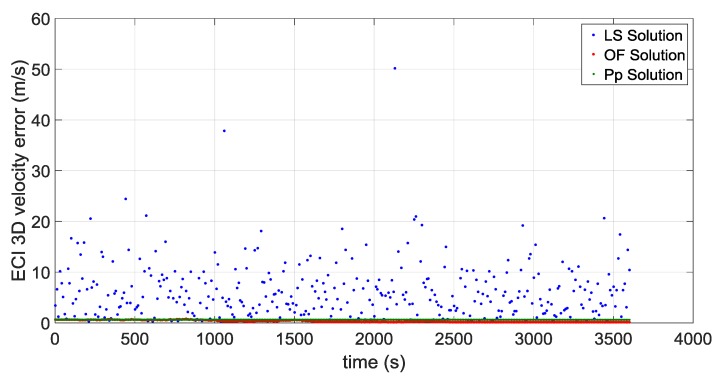
3D velocity error for the considered portion at Moon altitude for: the single-epoch least square (LS) solution (in **blue**); the orbital filtered (OF) solution (in **red**); and the propagated (Pp) solution (in **green**).

**Figure 21:**
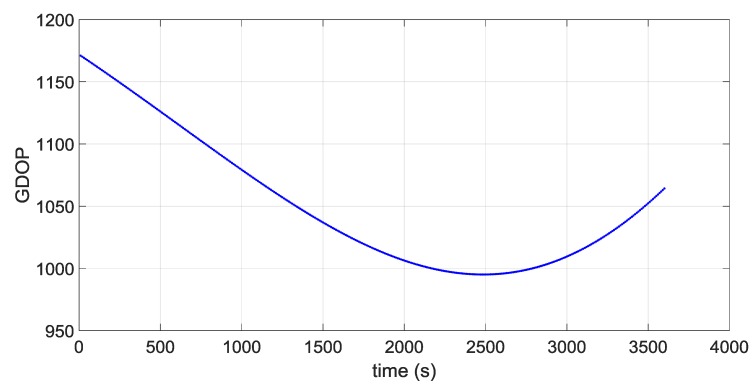
GDOP during the MTO portion at Moon altitude.

The first portion starts approximately at 36,000 km of altitude (altitude of the Geostationary Earth Orbit) and a second portion starts approximately at the average distance of the Moon from the centre of the Earth of 384,400 km, very close to the apogee of the MTO. Figure 16 and Figure 17 illustrate the ECI 3D position and velocity accuracy, respectively, of a least square (LS) single-epoch solution, a pure propagated (Pp) solution (obtained only propagating the initial condition through the orbital forces model), and the orbital filtered (OF) solution, which integrates by means of the developed adaptive EKF the LS together with the Pp solution. The LS solution is as expected very noisy due to the noise, which affects pseudorange measurements and it increases with the altitude because of the increasing GDOP (see Figure 18). On the other side, the Pp position solution has a significant drift due to the error in the initial condition; in fact, after about 250 s the position error reaches 50 m as expected because of the initial 0.2 m/s error in the initial velocity. (0.2 m/s × 250 s = 50 m). The OF solution is several times more accurate; the GPS measurements prevent the orbital propagation solution drifting, while the orbital propagation smooths the GPS solution. Figure 19, Figure 20 and Figure 21 illustrate the same quantities but for the MTO portion at Moon altitude. In this case as well, the benefit of the orbital filter is even more significant since the OF solution is more than one order of magnitude more accurate than the LS solution.

Note that the Hardware in the Loop (HIL) tests have not been carried out over the full MTO duration as it lasts almost 5 days and we currently do not have that simulation capability. Instead, the full MTO test has been performed in post processing by simulating pseudorange and pseudorange rate measurements (see Figures 10 and 11 in [26]) to assess the OF performance. Then, several HIL tests we performed for the same portion and for different potions of the full MTO allowed us to determine that a 1 h test duration is well enough to let the OF converge. Afterwards, we have performed HIL tests for several representative portions of 1 h durations to assess the acquisition, tracking, data decoding, and finally navigation performance (as reported in this section) of the *WeakHEO* receiver for the signals encountered in a lunar mission. It is also important also to keep in mind that the current *WeakHEO* receiver is only a proof of concept and thus not the final device ready to operate in a lunar mission, for which clearly more and longer tests will be required.

## 4. Conclusions

Following our previous feasibility studies of GNSS as navigation system to reach the Moon, this research paper describes the proof of concept of the GPS L1 C/A “*WeakHEO*” receiver for lunar mission, wholly developed in our laboratory in the last two years. After highlighting the characteristics of the GPS L1 C/A signals for the considered MTO, which have been identified in our previous research work, the requirements and constraints in the receiver design are defined. Afterwards, the general receiver architecture is described, providing a more detailed description of the acquisition, tracking and navigation modules. These modules are specifically designed for use with the dynamic environment and signal conditions seen in high altitude space applications, and the architecture presented is capable of performing acquisition, tracking, data synchronization and demodulation down to a level of 15 dB-Hz. This is verified on the hardware with tests using representative RF signals produced by a GNSS simulator. The navigation module makes use of an adaptive orbital filter, which exploits an orbital forces model to filter the noisy GPS least square single-epoch solutions and is able to significantly increase the navigation accuracy to a few hundred meters at Moon altitude. The filtered navigation solution can then be used to aid the acquisition and tracking modules allowing higher sensitivities also in high dynamics.

## 5. Future Work

The performance will be further improved in the new “*WeakHEO 2*” version of our receiver, where the available hardware resources will be increased, allowing the integration of the navigation module into the processor within the FPGA. This will allow the orbital filter to provide aiding to the acquisition and tracking modules at a high rate to improve performance as shown in [26]. The orbital filter of the “*WeakHEO 2*” receiver will directly filter the raw pseudorange and pseudorange rate observations instead of the single-epoch least square solution, with an expected higher navigation accuracy also in case of less than four GPS observations available. In addition, the iteration rate of the navigation output will be increased from 0.1 Hz to 1 Hz and the number of tracking channel increased from 6 to at least 12 channels. Furthermore, in the future we will also consider processing other frequencies and signals of other GNSS constellations to further improve the overall performance.

## Figures and Tables

**Table 1:** Initial position and velocity of the considered receiver trajectory and spacecraft parameters.

Parameters	Values
ECI Initial position (km)	$\left(\begin{array}{ccc} 2395.52 & -5298.28 & -3022.82 \end{array}\right)$
ECI Initial velocity (km/s)	$\left(\begin{array}{ccc} 10.19 & 3.58 & 1.72 \end{array}\right)$
Departure date	2 July 2005, 00:34:18
Mass of the spacecraft (kg)	1000
Reference surface (m^2^)	20
Radiation pressure coefficient	1

**Table 2:** Theoretical acquisition parameters.

Quantity	Value
C/N0 (dB-Hz)	15
Sampling rate (MHz)	4.096
Quantization (bit)	4
Quantization loss (dB)	0.05
Coherent integration time (ms)	20
Coherent gain (dB)	46
Frequency search step (Hz)	25
Worst case frequency mismatch loss (dB)	0.91
Code search step (chip)	0.25
Worst case code alignment loss (dB)	1.16
Data bit alignment loss (dB)	0.92
Squaring loss (dB)	5.73
Final desired SNR (dB)	16
Non-coherent gain required (dB)	26.77
Number of non-coherent integration	475
Total accumulation time (s)	9.5
Maximum tolerable Doppler rate error (Hz/s)	2.63
